# Identification of Benzyloxy Carbonimidoyl Dicyanide Derivatives as Novel Type III Secretion System Inhibitors *via* High-Throughput Screening

**DOI:** 10.3389/fpls.2019.01059

**Published:** 2019-09-05

**Authors:** Yi-Nan Ma, Liang Chen, Nai-Guo Si, Wen-Jun Jiang, Zhi-Gang Zhou, Jun-Li Liu, Li-Qun Zhang

**Affiliations:** ^1^Department of Plant Pathology and MOA Key Laboratory of Pest Monitoring and Green Management, China Agricultural University, Beijing, China; ^2^State Key Laboratory of the Discovery and Development of Novel Pesticide, Shenyang Sinochem Agrochemicals R&D Co., Ltd, Shenyang, China; ^3^China-Norway Joint Lab on Fish Gut Microbiota, Feed Research Institute, Chinese Academy of Agricultural Sciences, Beijing, China

**Keywords:** *Acidovorax*, *Xanthomonas*, *Pseudomonas*, type III secretion system, hypersensitive response, virulence inhibitor, benzyloxy carbonimidoyl dicyanide

## Abstract

The type III secretion system (T3SS) in many Gram-negative bacterial pathogens is regarded as the most critical virulence determinant and an attractive target for novel anti-virulence drugs. In this study, we constructed a T3SS secretion reporter containing the *β-*lactamase gene fused with a signal peptide sequence of the T3SS effector gene, and established a high-throughput screening system for T3SS inhibitors in the plant pathogenic bacterium *Acidovorax citrulli*. From a library of 12,000 chemical compounds, we identified a series of benzyloxy carbonimidoyl dicyanide (BCD) derivatives that effectively blocked T3SS-dependent *β-*lactamase secretion. Substitution of halogens or nitro groups at the para-position on the benzene ring contributed to an increased inhibitory activity. One representative compound, BCD03 (3,4-dichloro-benzyloxy carbonimidoyl dicyanide), dramatically reduced pathogenicity of *A. citrulli* on melon seedlings, and attenuated hypersensitive responses in the non-host *Nicotiana tabacum* caused by pathogenic bacteria *A. citrulli*, *Xanthomonas oryzae* pv. *oryzae* and *Pseudomonas syringae* pv. *tomato* at sub-MIC concentrations. Western blotting assay further confirmed that BCD03 inhibited effector secretion from the above bacteria *via* T3SS in the liquid medium. Taken together, our data suggest that BCD derivatives act as novel inhibitors of T3SS in multiple plant bacterial pathogens.

## Introduction

Antibiotic resistance is becoming a major concern in agricultural industries. Traditional antibiotics, which target growth and survival, induce high selective pressure on pathogenic bacteria. One promising way to reduce bacterial resistance is to target virulence factors, rather than growth (Rasko and Sperandio, 2010). The type III secretion system (T3SS) is highly conserved among Gram-negative bacteria and is directly related to pathogenicity during host cell invasion. The needle-like T3SS penetrates the cell membrane where it injects T3SS effectors (T3Es), suppressing host immunity and promoting invasion and dissemination (Cornelis, 2006). T3SS is not necessary for bacterial survival, making it an attractive target for novel antibacterial drugs (Büttner, 2012). To date, a number of chemical compounds acting as T3SS inhibitors have been identified in a wide range of important plant and animal bacterial pathogens, such as *Erwinia*, *Pseudomonas, Xanthomonas*, *Escherichia*, *Yersinia* and *Salmonella* species (Felise et al., 2008; Wang et al., 2011; Jessen et al., 2013; Yang et al., 2014; Anantharajah et al., 2016; Fan et al., 2017).

Most of the known T3SS inhibitors were screened out by using transcriptional reporters that fused T3SS-related genes to the genes encoding reporter proteins like luciferase, phospholipase, and *β*-lactamase. (Kauppi et al., 2003; Li et al., 2005; Felise et al., 2008; Pan et al., 2009; Duncan et al., 2014). A few screenings were performed by using translational or translocational reporters (Kim et al., 2009; Pan et al., 2009; Swietnicki et al., 2011). The known inhibitors, which include alicylates, benzoates, thiazoles, phenolic compounds and specific antibodies, affect T3SS functions by directly targeting T3SS injectisome (Lynch et al., 2010; Jessen et al., 2013; Bowlin et al., 2014), regulating T3SS gene expression (Yang et al., 2014; Marsden et al., 2015; Fan et al., 2017), or through other indirect interactions (Wang et al., 2011; Bowlin et al., 2014; Faure et al., 2014).

In many plant bacterial pathogens, T3SS and T3Es are essential virulence factors during infection. T3SSs in plant bacteria fall into two groups, hypersensitive reaction and pathogenicity (Hrp) groups I and II, based on their nucleotides, operon structures, and regulatory systems (Alfano and Collmer, 1996; Cornelis, 2006; Nilles and Condry, 2017). Representatives of group I include *Pseudomonas syringae* and *Erwinia amylovora*, while *Ralstonia solanacearum* and *Xanthomonas oryzae* belong in group II (Tampakaki et al., 2010; Diepold and Armitage, 2015). Although these two groups share similar *hrc* (HR and conserved) genes, some of their *hrp* genes appear completely different. One significant difference is that group I T3SSs are activated by HrpL, an ECF family sigma factor activated by HrpS to promote gene expression (Lidell and Hutcheson, 1994; Alfano and Collmer, 1996), while group II T3SSs are regulated by AraC family transcriptional regulators, HrpB in *R. solanacearum* and HrpX in *X. oryzae* and *Acidovorax citrulli*. *HrpB* and *hrpX* are activated by the OmpR-type transcriptional regulator HrpG, a response regulator protein of a two-component signal transduction system, which induces expression of T3SS (Wengelnik and Bonas, 1996; Wengelnik et al., 1996; Tampakaki et al., 2010). However, expression of T3SS is not constitutive. Transcription of T3SS genes in both Hrp groups is repressed in full nutrient medium, but induced *in planta* or in medium mimicking the intracellular space of plants (Bonas, 1994; Nilles and Condry, 2017). Induction of T3SS expression tends to involve low-nutrient culture media; for example, XVM2 and XOM2 medium for *X. oryzae* (pH 6.5 and 5 mM Mg^2+^), 1/4 M63 medium for *R. solanacearum* (1 mM Mg^2+^and 3.9 mM Fe^2+^) and MG medium for *P. syringae* (pH 7.0 and 2 mM Mg^2+^) (Keane et al., 1970; Boucher et al., 1985; Tsuge et al., 2002). Meanwhile, with bacteria such as *A. citrulli*, T3SS expression is induced in modified lysogenic broth (LB) medium (pH5.8, 10 mM Mg^2+^) (Zhang et al., 2018).

*A. citrulli* is the causal agent of bacterial fruit blotch in cucurbit crops such as watermelons (*Citrullus lanatus*) and melons (*Cucumis melo*) (Schaad et al., 2008). T3SS is required for pathogenicity and hypersensitive response (HR) induction in host and non-host plants, respectively (Bahar and Burdman, 2010; Johnson et al., 2011). In this study, we established a high-throughput screening system for T3SS inhibitors in *A. citrulli* strain MH21 and successfully screened a large number of chemical compounds under the optimized T3SS-inducing conditions. Chemicals showing inhibitory activity were further tested using different plant bacterial pathogens to explore their effective target spectrum.

## Materials and Methods

### Bacterial Strains, Plasmids, and Growth Conditions

The bacterial strains and plasmids used in this study are listed in **Table 1**. *Escherichia coli* was grown in LB medium at 37°C, *A. citrulli* MH21 and its derivatives were grown in LB medium at 28°C, *X. oryzae* pv. *oryzae* PXO99^A^ and its T3SS mutant were grown in M210 medium (0.8% casein enzymatic hydrolysate, 0.5% sucrose, 0.4% yeast extract, 17.2 mM K_2_HPO_4_, 1.2 mM MgSO_4_·7H_2_O) at 28°C. XOM2 medium (0.18% D-(+) xylose, 670 μM L-methionine, 10 mM sodium L-(+) glutamate, 14.7 mM KH_2_PO_4_, 40 μM MnSO_4_, 240 μM Fe(III)-EDTA and 5 mM MgCl_2_, pH adjusted to 6.5 with KOH) was used for *hrp*-inducing (Tsuge et al., 2002). *P. syringae* pv. *tomato* DC3000 and its T3SS mutant were grown in King’s B medium at 28°C. MG medium (1% Mannitol, 0.2% L-glutamic acid, 3.6 mM KH_2_PO_4_, 3.4 mM NaCl and 1.6mM MgSO_4_·7H_2_O, pH adjusted to 7.0 with NaOH) was used for *hrp*-inducing (Keane et al., 1970). The following antibiotics were used when required: 50 μg/mL ampicillin (Ap), 50 μg/mL streptomycin (Sm), 25 μg/mL spectinomycin (Sp), 50 μg/mL kanamycin (Kan), 10 μg/mL gentamycin (Gm), and 50 μg/mL rifampicin (Rif).

**Table 1:** Bacterial strains and plasmids used in this study.

Strain or plasmid	Relevant characteristics^a^	Reference or source
Strain		
*Escherichia coli*		
DH5α	F^−^ *recA1 endA1 hsdR17 supE44 thi-1 gyrA96 relA1Δ (argFlacZYA) I169*Φ*80lacZ ΔM15*	Invitrogen
*Acidovorax citrulli*		
MH21	Wild-type, Ap^R^	Ren et al. (2014)
MH21Δ*hrcC*	*hrcC* in MH21 mutated; Ap^R^, Rif^R^	Lab collection
MH21Δ*penAC*	*penAC* in MH21 mutated; Rif^R^	This study
MH21Δ*penAC*Δ*hrcC*	*hrcC* and *penAC* double mutant; Rif^R^	This study
*X. oryzae* pv. *oryzae*		
PXO99^A^	Wild-type strain, Philippine race 6	Hopkins et al. (1992)
PXO99^A^Δ*hrcU*	*hrcU* in PXO99^A^ mutated	Liu et al. (2010)
*P. syringae* pv. tomato		
DC3000	Wild type; Rif^R^	Buell et al. (2003)
DC3000Δ*hrcQ-U*	*hrcQ-U* in DC3000 mutated; Rif^R^	Badel et al. (2006)
		
Plasmids		
pBBR1MCS-2	pBBR1MCS-2 containing pBluescript II KS-*lacZ*α; Km^R^	Kovach et al.(1994)
pRK600	Helper plasmid; ColE1 replicon TraRK^+^ Mob^+^	Finan et al. (1986)
pZLQ-Flag	Expression vector; Km^R^	Luo and Farrand (1999)
pZAC	Expression vector; Km^R^	This study
pZAC-3502sig-*penAC*	Expression vector pZAC carrying *penAC* fused with N-signal peptide sequence of a T3SS effector; Km^R^	This study
pZAC-3502-FLAG	Expression vector pZAC carrying the full-length *hopAO1* gene with a C-FLAG tag; Rif^R^, Km^R^	This study
pHZY-*avrXa27*	Expression vector pHM1 carrying *avrXa27* with a C-FLAG tag; Ap^R^, Sp^R^	Ji et al. (2014)
pCPP5372-*avrPto*-HA	Expression vector pCPP5372 carrying *avrPto* with a C-HA tag; Gm^R^	Wei et al. (2015)

a ApR, CmR, KmR, RifR, SpR and GmR indicate resistance to ampicillin, chloramphenicol, kanamycin, rifampin, spectinomycin and gentamycin, respectively.

### Sources of Screened Compounds

Chemical compounds used for T3SS inhibitor screening were provided by Shenyang Sinochem Agrochemicals R&D Co. Ltd (Shenyang, China) and were dissolved in dimethyl sulfoxide (DMSO).

### Construction of the T3SS Secretion Reporter Strain

To construct the T3E secretion reporter pZAC-3502sig-*penAC*, the *penAC* (GenBank accession MK576102) fragment without its 57-bp 5’ Type II secretion system (T2SS) signal peptide sequence was PCR amplified from MH21 using primers *penAC*-F and *penAC*-R (**Table S1**). A 180-bp T3SS-dependent signal peptide sequence from the predicted effector *Aave_3502*, a homologue of *P. syringae* effector HopAO1 (Bretz et al., 2003; Underwood et al., 2007), was PCR amplified using primers Aave-3502sig-F and Aave-3502sig-R (**Table S1**) then in-frame fused to the 5’end of the *penAC* fragment digested with *Eco*RI. The modified *β*-lactamase sequence was cloned into pZLQ (Luo and Farrand, 1999) under control of the P_trc_ promoter. The reporter plasmid was introduced from *E. coli* into *A. citrulli* MH21 *via* mating with an *E. coli* strain carrying the helper plasmid (pRK600) (Finan et al., 1986). The pZAC-3502-FLAG plasmid containing the FLAG-tagged *hopAO1* gene under the P_trc_ promoter was generated and introduced into *A. citrulli* MH21 for Western blotting analysis. The *X. oryzae* pv. *oryzae* carrying plasmid pHZY-*avrXa27* (Ji et al., 2014) and *P. syringae* pv. *tomato* DC3000 carrying plasmid pCPP5372-*avrPto*-HA (Wei et al., 2015) were also used for Western blotting analysis.

### High-Throughput Screening of T3SS Inhibitors

*A. citrulli* MH21 reporter strain containing the pZAC-3502sig-*penAC* plasmid was grown overnight in LB medium at 28°C then the bacterial cells were harvested by centrifugation. After washing twice with sterilized distilled water (sdH_2_O), the bacterial cells were resuspended in sdH_2_O to OD_600_ = 1.0 and inoculated into LB medium (pH 5.8) supplemented with 10 mM MgCl_2_ (Zhang et al., 2018) in a 96-well microplate at a ratio of 4%. Test compounds were added at a final concentration of 25 μg/mL. An identical concentration of DMSO was used as a control. Nitrocefin (TOKU-E, WA, USA) was added at a concentration of 250 μg/mL when the bacteria grew to OD_600_ = 0.4–0.6 (20 h) followed by incubation for 15 min. Color measurements of the medium (OD_500_) were then taken using a Microplate Reader (SpectraMax i3x; Molecular Devices, Sunnyvale, CA). The screening experiment was performed with three technical replicates.

### Minimum Effective Concentration (MEC) Assay and Half Maximal Inhibitory Concentration (IC50)

Bacterial strain MH21 harboring the pZAC-3502sig-*penAC* plasmid was prepared as above in a 96-well microplate. The MEC assay was carried out by diluting benzyloxy carbonimidoyl dicyanide (BCD) derivatives at a final concentration of 70, 60, 50, 40, 30, 20, 10, 5, 2.5, or 1.25 μg/mL, respectively. Nitrocefin was then added and the OD_500_ was determined as described above. The molarity of chemical compounds was converted based on the MECs and molecular weights (MWs). IC50 of these compounds was calculated based on the OD500 values and molarity (Sebaugh, 2011). Experiments were performed twice with three biological replicates.

### Minimum Inhibitory Concentration (MIC) Assay

A MIC assay was carried out using strains MH21, PXO99^A^ and DC3000 and their T3SS mutants. Bacteria were cultured in their own favorable medium until OD_600_ reached 1.0 then inoculated in 96-well microplates with the same medium at a ratio of 1:1,000, supplemented with BCD derivatives at a final concentration of 200, 100, 50, 25, 12.5, 6.25 or 3.125 μg/mL, respectively. The microplates were placed at 28 ºC and OD_600_ was measured using a microplate reader after 48 h incubation. The experiments were performed twice with three biological replicates.

### Pathogenicity Inhibition Assay

Germinated seeds of melon (*Cucumis melo* INV192) were dipped in MH21 bacterial suspension at 3 × 10^8^ CFU, supplemented with 25 or 50 μg/mL BCD03 for 20 min. Δ*hrcC* mutant strain was used as a control. Cotyledons of the seedlings were collected 12 days after sowing and disease severity was assessed on a scale of 1 to 9: 1, no symptoms; 3, small, necrotic lesions on cotyledons, <20% necrotic cotyledon; 5, necrotic lesions with chlorosis on cotyledons, 20–50% necrotic cotyledon; 7, large spreading lesions, >50% of cotyledon necrotic; 9, >90% necrosis of the cotyledon or a dead plant (Hopkins and Thompson, 2002). The experiments were repeated twice with three biological replicates. Each replicate contained 5 seedlings.

### Hypersensitive Response Assays

*Nicotiana tabacum* seedlings were grown under greenhouse conditions for 4–5 weeks. Bacterial cells were then harvested from precultured medium and suspended in 10 mM MgCl_2_. Leaves of *N. tabacum* were subsequently inoculated with bacterial strains at 3 × 10^8^ cfu/mL (MH21, PXO99^A^ and their T3SS mutants) or 3 × 10^6^ cfu/mL (DC3000 and its T3SS mutant) by infiltration with a blunt syringe and assayed 18 h post inoculation (Alfano et al., 1996). BCD03 was added at a final concentration of 25 μg/mL 2 h before infiltration. BCD03 alone was used as a control. The experiment was performed with three biological replicates.

### Effector Secretion and Immunodetection

Strains MH21, PXO99^A^ and DC3000 harboring reporter plasmids pZAC-3502-FLAG, pHZY-*avrXa27* and pCPP5372-*avrPto*-HA, respectively, were cultured in their own *hrp*-inducing medium supplemented with 25 μg/mL T3SS inhibitor BCD03 (or DMSO as a control) at 28ºC for 8 h. Bacterial cultures were diluted to OD_600_ = 0.5 and centrifuged at 4000 × g for 10 min then the supernatant was filter sterilized, mixed with cold 100% trichloroacetic acid to a final concentration of 15% and incubated overnight at 4ºC. Samples were then centrifuged again at 6000 × g for 30 min at 4ºC, the supernatant was discarded, and the protein pellet was washed twice with 2 mL cold acetone. The bacterial pellet was resuspended in 50 µl phosphate buffered saline (PBS) and boiled for 10 min then HopAO1 and AvrXA27 were detected by Western blotting using a primary anti-FLAG mouse monoclonal antibody (Transgen Biotech, Beijing, China), diluted 1:2,000, and a goat anti-mouse IgG (H+L) secondary antibody conjugated to HRP (Invitrogen, Beijing, China), diluted 1:5,000. AvrPto was detected by Western blotting using a primary anti-HA mouse monoclonal antibody conjugated to HRP (MBL, Beijing, China) and diluted 1:5,000. Antibodies used in this study were all diluted in 40 ml PBS buffer supplemented with 0.5% tween 20 and 1% skimmed milk. Immunodetected HopAO1-FLAG, AvrXA27-FLAG, and AvrPto-HA were developed using Immobilon ECL (Beyotime, Shanghai, China) and membranes were photographed using the ChemiDoc Imaging System (BIO-RAD, Beijing, China).

### Statistical Analyses

Statistical analyses were performed using IBM SPSS Statistics 17.0 (SPSS, Chicago, IL, USA) or GraphPad 7.0 (GraphPad Software, La Jolla, CA, USA) as specified.

## Results

### Development of a T3SS Inhibitor Screening System in *Acidovorax citrulli*

**Figure 1:**
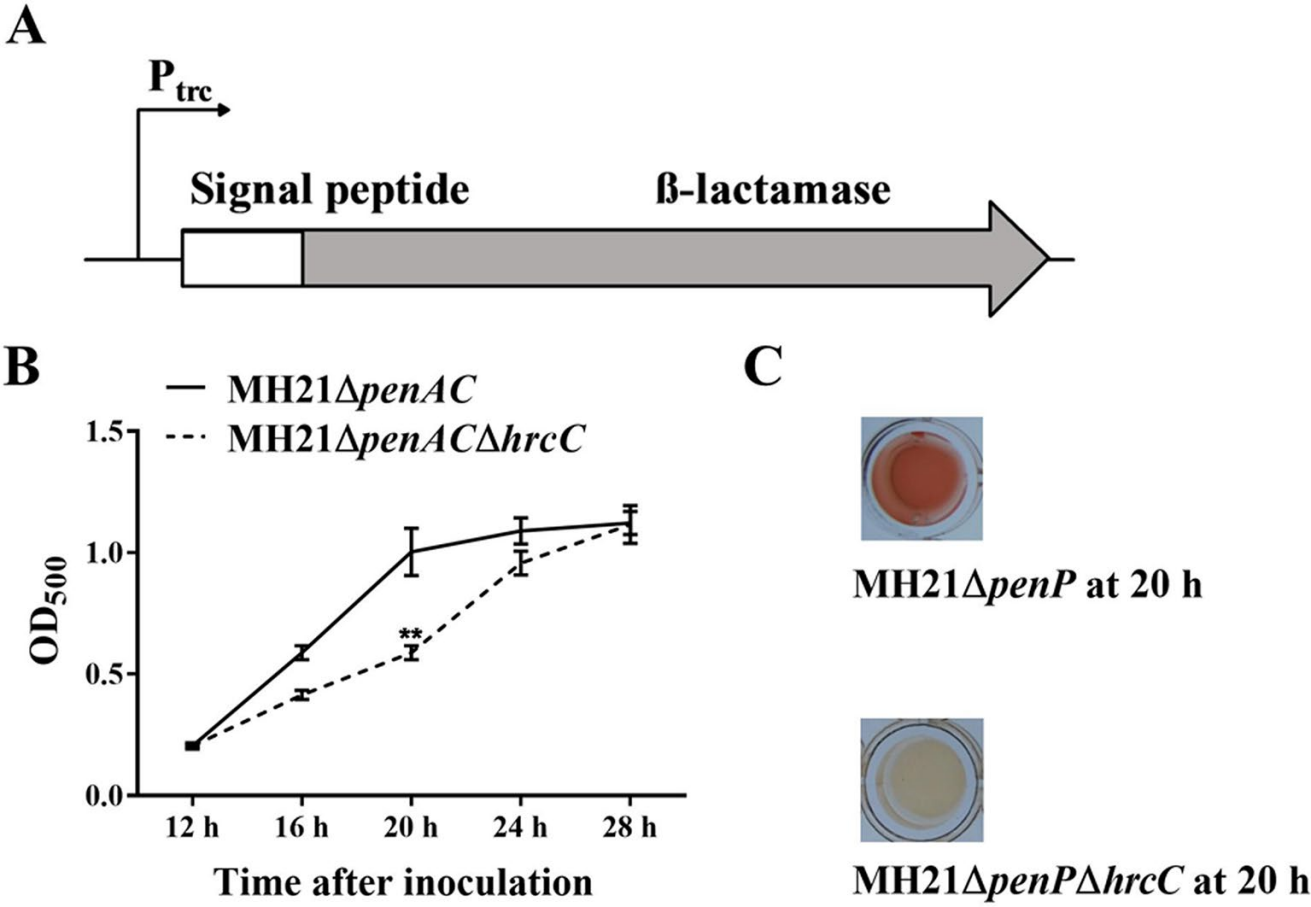
Reporter structure and suitable growth state of reporter strain *Acidovorax citrulli* MH21∆*penAC* (pZAC-3502sig-*penAC*). **(A)** Reporter structure on plasmid pZAC-3502sig-*penAC*. The P_trc_ promoter driven *β*-lactamase gene was fused with a signal peptide sequence of the T3SS effector gene (*Aave_3502*, a *hopAO1* homologous gene). **(B)** Extracellular *β*-lactamase activity of MH21*∆penAC* and its T3SS mutant MH21∆*penAC*∆*hrcC* cultured in *hrp*-inducing medium. *β*-lactamase activity was measured spectrophotometrically by determining the OD_500_. Experiments were performed twice with three biological replicates. Statistically significant differences (Student’s t-test) were observed at 20 h post inoculation. P-value < 0.05 (**). **(C)** The reaction mixture containing the reporter plasmid in the T3SS mutant MH21∆*penAC*∆*hrcC* remained yellow, but changed into red in mixture containing the T3SS wild-type strain MH21∆*penAC*.

We constructed a T3E secretion reporter plasmid containing a T3E signal peptide sequence fused with the *β*-lactamase gene in *A. citrulli* MH21 to screen for T3SS inhibitors. The *β*-lactamase gene was used as a reporter because it hydrolyzes nitrocefin and gives a clear color change readout from yellow to red, fulfilling the purpose of high through-put screening. However, wild-type *A. citrulli* MH21 is naturally resistant to ampicillin and hydrolyzes nitrocefin, indicating the presence of a gene with *β*-lactamase activity on its chromosome (**Figure S1A**). Genome sequence analysis subsequently revealed a potential *β*-lactamase gene named *penAC* in MH21, which had 39.8% identity and 83.0% coverage with the *β*-lactamase gene *penP* in *Bacillus subtilis* (GenBank accession BG11016). Further phylogenetic analysis (**Figure S2**) and conserved structure analysis (**Table S2**) revealed that PenAC belongs to *β*-lactamase class A (Ambler, 1980; Matagne et al., 1999; Eiamphungporn et al., 2018). In addition, mutation of *penAC* in *A. citrulli* MH21 led to loss of ampicillin resistance (**Figures S1A, B**), suggesting that PenAC is indeed a *β*-lactamase. We therefore constructed pZAC-3502sig-penAC, a reporter plasmid containing the Ptrc promoter to drive a recombined *β*-lactamase gene *penAC*. The original N-terminal T2SS signal peptide of *penAC* was replaced by the T3SS-signal peptide of effector gene *Aave_3502*, a homologue of the *P. syringae* effector HopAO1. (**Figure 1A**). The mutant MH21Δ*penAC* was then used as a host for the reporter plasmid pZAC-3502sig-*penAC* to remove the background *β*-lactamase activity. A double gene mutant, MH21Δ*penAC*Δ*hrcC*, was also constructed as a T3SS-negative control (**Figure S1C**).

When cultured in T3SS-inducing LB medium (pH 5.8, 10 mM MgCl_2_), MH21Δ*penAC* containing the reporter plasmid pZAC-3502sig-*penAC* secreted *β*-lactamase in a T3SS-dependent manner. Extracellular *β*-lactamase activity was therefore used to indicate the effect of each inhibitor. Because over-cultured bacteria release undesired *β*-lactamase *via* cell lysis, we identified the most suitable bacterial state for reporter detection by comparing extracellular *β*-lactamase activity between the wild-type MH21 and its T3SS mutant throughout growth. As a result, no growth differences were observed between MH21 mutants Δ*penAC* and Δ*penAC*Δ*hrcC* carrying pZAC-3502sig-*penAC* when cultured in T3SS-inducing medium for 28 h. However, extracellular *β*-lactamase activity was significantly higher with MH21Δ*penAC* than the T3SS mutant MH21Δ*penAC*Δ*hrcC* at 20 h (OD_600_ = 0.4– 0.6) post inoculation (**Figures 1B, C**), indicating secretion of *β*-lactamase *via* T3SS. These culture conditions were therefore used to screen the T3SS inhibitors.

### Benzyloxy Carbonimidoyl Dicyanide as a Novel Inhibitor of Bacterial T3SS

**Figure 2:**
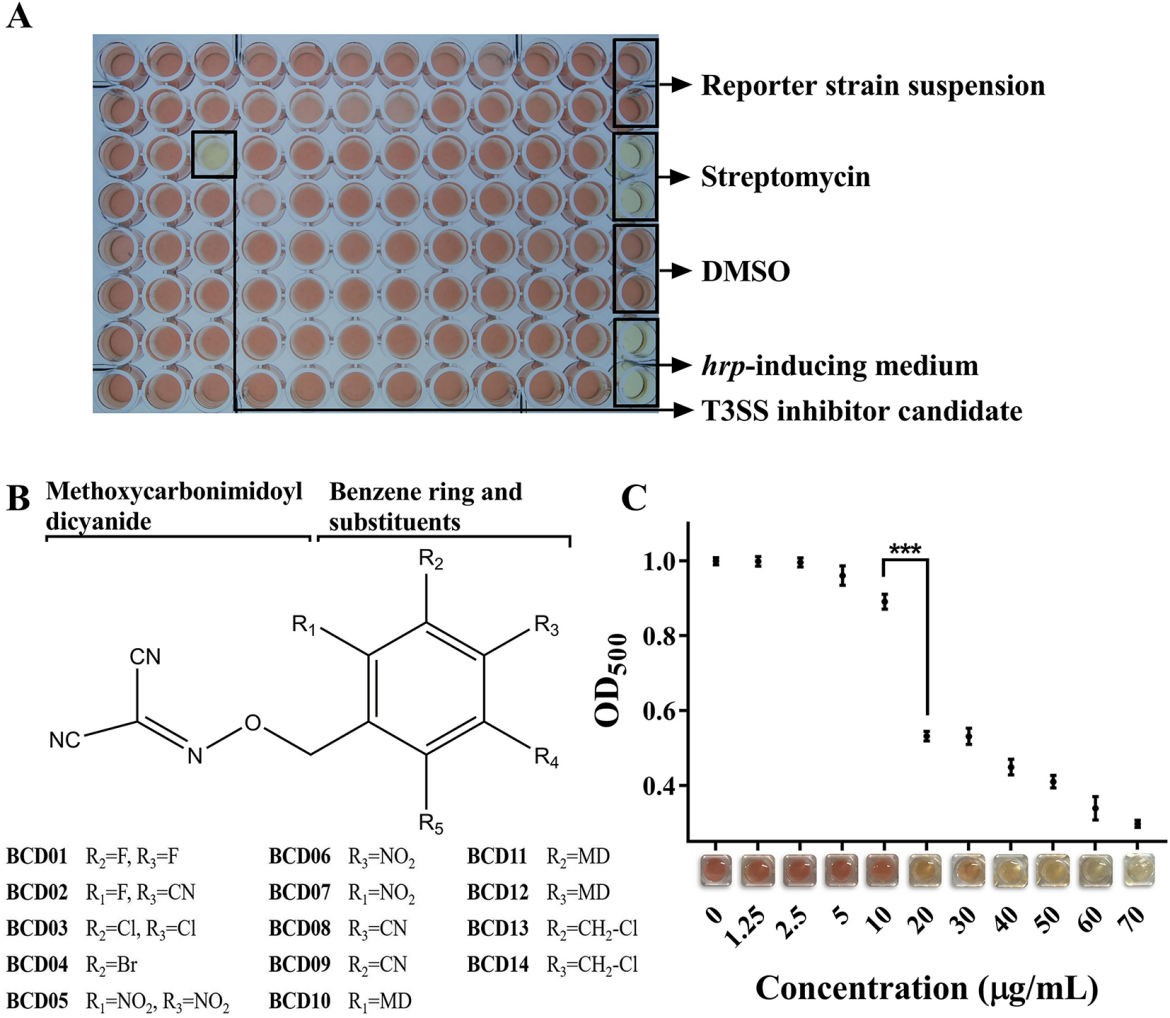
Benzyloxy carbonimidoyl dicyanide derivatives (BCDs)-induced inhibition of T3SS function in *Acidovorax citrulli* MH21. **(A)** High-throughput screening in a 96-well microplate. Each well contained the reporter strain MH21∆*penAC* harboring pZAC-3502sig-*penAC* in 200 µL *hrp*-inducing medium (LB with 10 mM MgCl_2_, pH 5.8) supplemented with candidate chemicals and nitrocefin. Control groups were arranged in the rightmost row, including reaction mixture without candidate chemicals, reaction mixture with the antibiotic streptomycin, reaction mixture with solvent DMSO, and *hrp*-inducing medium only. **(B)** Chemical structures of BCD derivatives that had an inhibitory effect on T3E reporter secretion. The benzene ring substituted by one BCD is stabilized as a core. R1-R5 substitutions on the benzene ring were evaluated. **(C)** Inhibitory effect of different concentrations of BCD03 on T3SS function. The readout of OD_500_ decreased as the concentration of BCD03 increased, as shown by the color variation from red to yellow. Experiments were performed twice with three biological replicates. Statistically significant differences (Student’s t-test) were observed between 10 and 20 µg/mL. P-value < 0.01 (***).

More than 12,000 compounds at a final concentration of 25 µg/mL were screened in 96-well microplates using the above method (**Figure 2A**). A series of BCD derivatives prevented the color change from yellow to red in the reaction mixture, indicating potential T3SS inhibitory activity. These compounds shared the same BCD backbone as a number of substituents, including halogen, hydro, nitro, and methoxycarbonimidoyl dicyanide (MD), on its benzene ring (**Figure 2B**). The MICs of the BCD derivatives was always higher than 50 µg/mL, and with BCD03, BCD10, and BCD12 was as high as 100 µg/mL (**Table S3**). These findings indicate specific inhibition of T3SS by these compounds. The BCD derivatives were further tested with the wild- type MH21, which produced T2SS-dependent *β*-lactamase PenAC using the system described above. Accordingly, no color variation was observed, indicating that the compounds did not directly inhibit *β*-lactamase activity (data not shown).

### Structure-Function Relationship of the BCD Derivatives

The MECs of effective inhibitors of T3SS from *A. citrulli* MH21 were subsequently measured at concentrations below their MICs. BCD01, BCD02, BCD03, BCD04, and BCD05 had relatively low MEC (**Table 2**). Moreover, using BCD03 as an example, the color of the reaction changed from red to yellow with increasing addition, indicating a dosage effect of the inhibition on the function of T3SS (**Figure 2C**).

**Table 2:** Derivatives of benzyloxy carbonimidoyl dicyanide (BCD) with their minimum effective concentrations (MEC), molecular weights (MW) and half maximal inhibitory concentrations (IC50).

	R^1^	R^2^	R^3^	MEC (µg/mL)	MW	IC_50_ (µM)
BCD01	–	F	F	20.0	221.2	57.5 ± 3.8
BCD02	F	–	CN	20.0	228.2	79.8 ± 6.9
BCD03	–	Cl	Cl	20.0	254.1	67.3 ± 2.5
BCD04	–	Br	–	20.0	264.1	52.1 ± 5.3
BCD05	NO_2_	–	NO_2_	20.0	275.2	47.6 ± 0.7
BCD06	–	–	NO_2_	30.0	230.2	90.0 ± 4.5
BCD07	NO2	–	–	40.0	230.2	134.0 ± 8.8
BCD08	–	–	CN	30.0	210.2	100.9 ± 8.1
BCD09	–	CN	–	30.0	210.2	121.1 ± 5.4
BCD10	MD	–	–	50.0	292.3	153.6 ± 7.6
BCD11	–	MD	–	50.0	292.3	149.7 ± 9.8
BCD12	–	–	MD	40.0	292.3	117.7 ± 9.4
BCD13	–	CH_2_–Cl	–	50.0	233.7	191.2 ± 7.6
BCD14	–	–	CH_2_–Cl	40.0	233.7	163.3 ± 8.2

R^1^–R^3^: ortho-, meta- and para-position of the substitutional group on the benzene ring. F, Cl, Br: halogen substitutional group. CN: cyan group. NO_2_: nitro group. “–”: no substitutional group. Experiments were performed twice with three biological replicates.

Benzene substituted by halogens as in BCD01, BCD02 (containing an additional cyan group), BCD03 and BCD04, had relatively low IC50 values that mean the high T3SS-inhibitory activities. BCD13 and BCD14 also possessed a halogen substitution, but not directly on the benzene, resulting in a decreased inhibitory activity. Of the three nitro-substituted BCDs (BCD05, BCD06, and BCD07), BCD05, which had two nitro substitutes at both a para- and ortho-position, had the highest inhibitory activity, suggesting that two substitutions are better than one. The increased inhibitory activity of BCD06 compared to BCD07 further suggests a para-position substitution is also beneficial under single substitutional situation. A similar position-activity relationship was observed with BCD10-12, whereby ortho-, meta- and para-position substitutions resulted in IC50 values of 153.6, 149.7, and 117.7 μM, respectively. However, cyan substitutions at para- and meta-positions did not result in any significant differences between BCD08 and BCD09. Overall, with the single substitution, para-position of the compounds resulted in the highest T3SS inhibitory activity, and the replacement of halogens or nitro substitutional groups resulted in an increase of T3SS inhibitory effect.

### BCD03 Suppresses the HR in *Nicotiana tabacum* and the Bacterial Pathogenicity in Melon Seedlings

**Figure 3:**
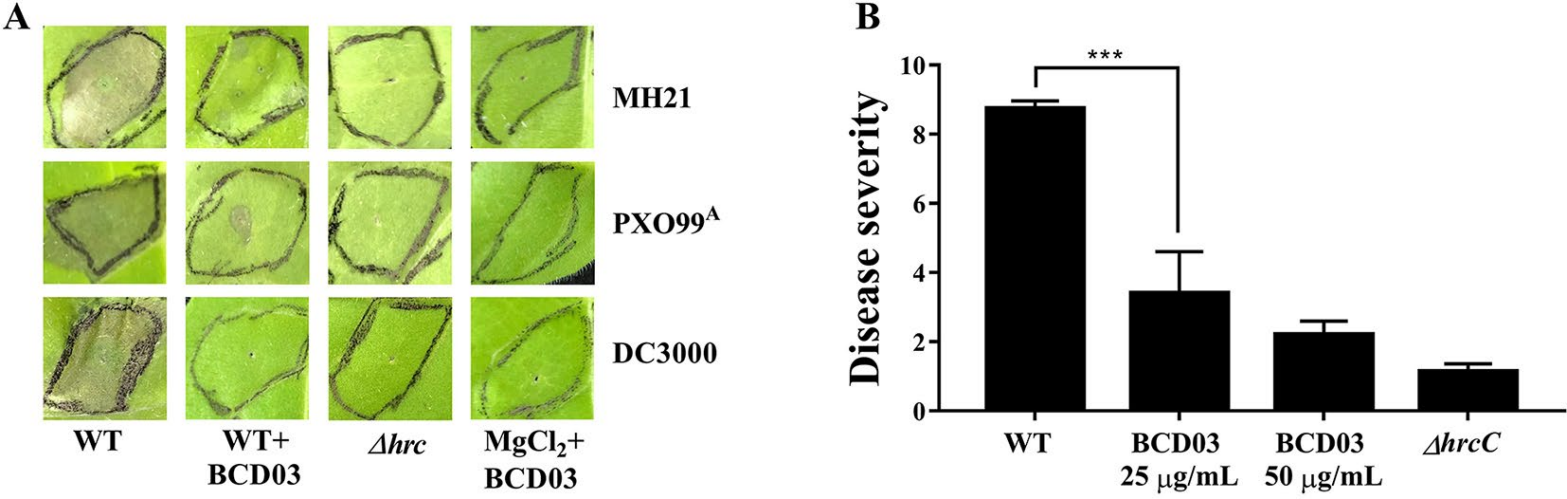
**(A)** BCD03 suppression of hypersensitive responses in *Nicotiana tabacum* triggered by pathogenic bacteria. *A. citrulli* MH21, *X. oryzae* pv. *oryzae* PXO99^A^, *P. syringae* pv. tomato DC3000 and their T3SS mutants, with or without BCD03 (25 µg/mL added 2 h before infiltration), were infiltrated into the leaves (3 × 10^8^ cfu/mL for MH21 and PXO99^A^, 3 × 10^6^ cfu/ml for DC3000) then the response was photographed at 18 h post inoculation. The control group was treated with 10 mM MgCl_2_ mix with 25 µg/mL BCD03. Photos from one of three biological replicate experiments are shown. **(B)** Effect of BCD03 on infection of *A. citrulli* MH21 on melon seedlings. Germinated melon seeds were dipped in bacterial suspension at 3 x 10^8^ CFU, supplemented with 0, 25 or 50 µg/mL BCD03, respectively. Disease severity was investigated 12 days after sowing. Disease ratings are defined using a scale-based symptom appearance as described in Methods section. The experiments were repeated twice with three biological replicates. Each replicate contained 5 seedlings. Statistically significant differences (Two-way ANOVA) were observed between wild type treated groups and other groups. P-value < 0.01 (***).

The HR-inducing capacities of bacteria belonging to Hrp groups I (*P. syringae* pv. tomato DC3000) and II (*X. oryzae* pv., *oryzae* PXO99^A^, and *A. citrulli* MH21), and their T3SS mutants were examined in *N. tabacum* in the presence of BCD03. MICs of BCD03 in strains DC3000, PXO99^A^, and MH21 were 200, 50 and 100 µg/mL, respectively (**Figure S3**). BCD03 was therefore used at the sub-MIC concentration of 25 µg/mL for all the above bacteria. HR symptoms were observed on leaves of *N. tabacum* 18 h after inoculation with wild-type bacteria (MH21, PXO99^A^, and DC3000), but were dramatically suppressed when BCD03 was added (**Figure 3A**). No cell death symptoms developed on leaves treated with the T3SS mutants or BCD03 alone (**Figure 3A**). These results suggest that BCD03 inhibits T3SS-based HR induction in bacteria of at least three genera belonging to two Hrp groups.

To test the inhibitory activity of BCD03 on bacterial pathogenicity, the wild type *A. citrulli* MH21 with or without BCD03 at its sub-MICs was inoculated on the germinated melon seeds before sowing. Disease investigation at seedling stage showed a sever disease caused by the wild type MH21, but not its T3SS mutant Δ*hrcC*, indicating the critical role of T3SS in bacterial infection (**Figure 3B**). A dramatic decrease of disease severity was observed on seedlings treated with the wild type MH21 and BCD03 at concentrations 25 µg/mL and 50 µg/mL (**Figure 3B**). As a result, BCD03 reduced the T3SS-dependent pathogenicity of *A. citrulli* MH21 on its host plant at sub-MICs.

### BCD03 Inhibits T3SS Effector Secretion

**Figure 4:**
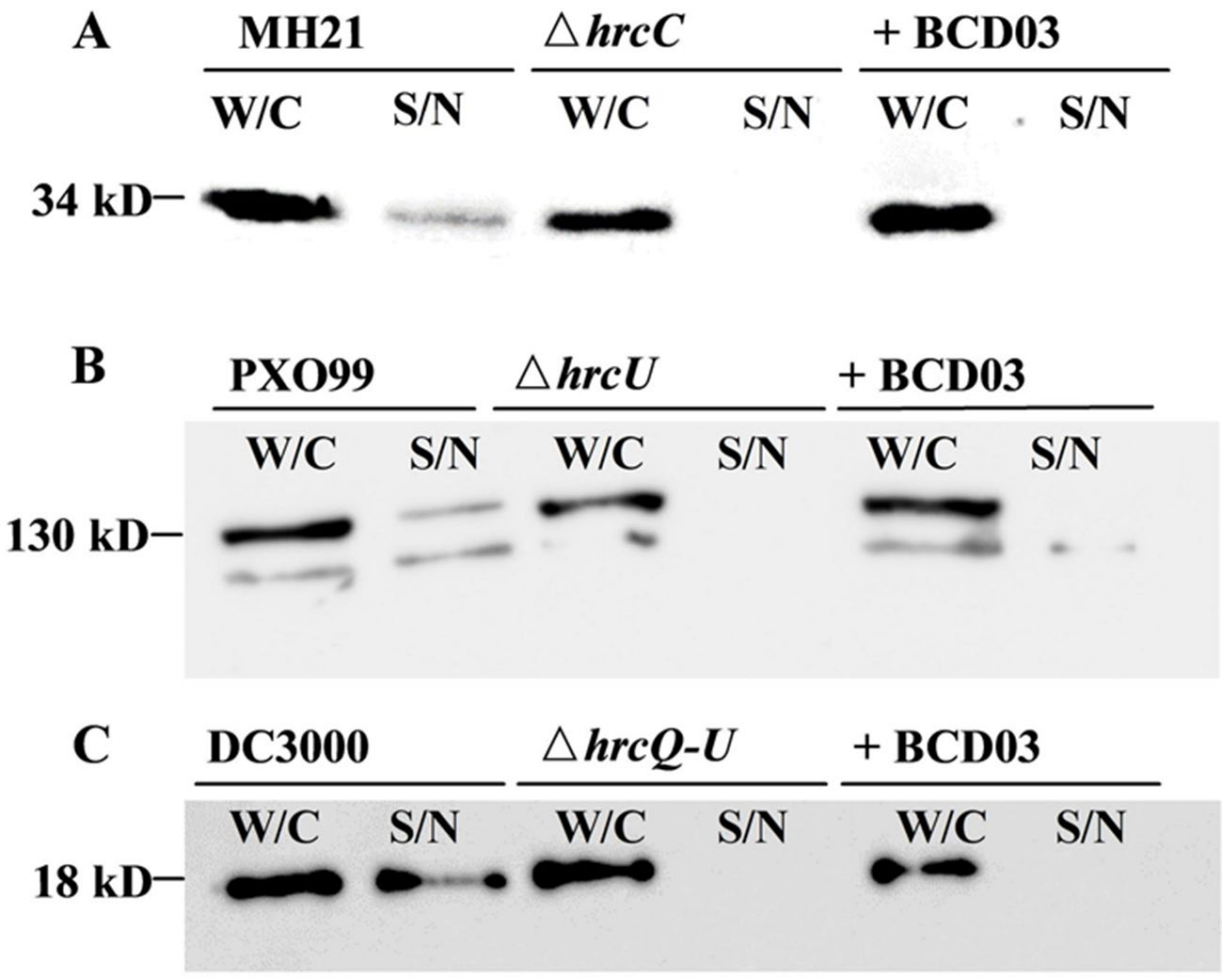
BCD03 inhibition of effector secretion *via* bacterial T3SS. **(A)** *A. citrulli* MH21 bearing the *hopAO1*-FLAG construct. **(B)** *X. oryzae* pv. *oryzae* PXO99^A^ bearing the *avrXa27*-FLAG construct. **(C)** *P. syringae* pv. *tomato* DC3000 bearing the *avrPto*-HA construct. All bacteria were grown for 8 h in their own *hrp*-inducing media with or without BCD03 at a finial concentration of 25 µg/mL. Whole cell extracts (W/C) and supernatants (S/N) were separated by centrifugation, and their secreted proteins were detected with anti-FLAG or anti-HA antibodies.

When cultured in *hrp*-inducing media, plant pathogenic bacteria deliver T3Es to extracellular spaces *via* T3SS. We therefore examined the inhibitory effect of BCD03 on T3SS-dependent effector secretion in three different bacteria, *A. citrulli* MH21, *X. oryzae* PXO99^A^, and *P. syringae* DC3000. All three wild-type bacteria and their T3SS mutants produced T3Es inside the cell, but only those of the wild-type bacteria were detectable in intracellular medium by Western blotting analysis, indicating the requirement of T3SS for effector secretion (**Figure 4**). BCD03 treatment suppressed secretion of effector HopAO1 and most of effector AvrXa27 in the Hrp group II bacterial strains MH21 and PXO99^A^, respectively (**Figures 4A, B**). Similarly, secretion of effector AvrPto was suppressed in Hrp group I strain DC3000 (**Figure 4C**). It is remarkable that intracellular production of T3Es in all three bacteria was not reduced, which suggests that only secretion but not expression of T3Es was inhibited by BCD03. Overall, the Western blotting results suggest that BCD03 has a broad-spectrum inhibitory effect on plant pathogen bacterial T3SSs.

### Discussion

In this study, we developed a high-throughput system based on a T3E secretion reporter to screen bacterial T3SS inhibitors, which is distinguished from most previous studies using transcriptional reporters of T3SS structure genes, regulatory genes or T3E genes (Kauppi et al., 2003; Gauthier et al., 2005; Li et al., 2005; Nordfelth et al., 2005; Pan et al., 2007; Felise et al., 2008; Pan et al., 2009; Aiello et al., 2010; Enquist et al., 2012; Fan et al., 2017). Inhibitors obtained from previous screening commonly affect transcription of T3SS structural gene clusters and T3E genes (Gauthier et al., 2005; Lee et al., 2007; Arnoldo et al., 2008; Kim et al., 2009; Garrity-Ryan et al., 2010; Grier et al., 2010; Swietnicki et al., 2011; Yamazaki et al., 2012; Kim et al., 2014; Williams et al., 2015; Marsden et al., 2016), while potential inhibitors targeting post-transcriptional regulation or effector secretion were missed. In this study, we constructed a T3E secretion reporter for inhibitor screening since effector secretion is the end result of T3SS expression, which is delicately regulated by a complex network, including transcriptional and post-transcriptional regulation of T3SS components and regulatory genes, post-translational modification of T3SS structural proteins and assembly of the needle or pilus structure, as well as interactions between T3Es and T3SS structural proteins (Keyser et al., 2008; Harmon et al., 2010; Kimura et al., 2011; Warrener et al., 2014). A similar screening system was developed in *Yersinia* using *β*-lactamase fused with the T3E YopE, revealing a group of compounds that specifically inhibited translocation of effectors into the host cells without altering the synthesis and secretion of T3Es (Harmon et al., 2010). Revealing T3SS inhibitors with this kind of action model is difficult by using transcriptional reporters, highlighting the importance of secretion reporters in large-scale screening.

Because our screening system detected T3SS-induced extracellular activity of *β*-lactamase, cell growth had to be tightly controlled to avoid *β*-lactamase leakage during bacterial cell lysis. With *A. citrulli* MH21, a narrow window of time was revealed when the bacteria were grown in suitable medium for 20 h (OD_600_ = 0.4–0.6), during which extracellular *β*-lactamase activity was significantly higher in the wild-type strain than its T3SS mutant. This difference was less obvious with further incubation, suggesting that *β*-lactamase release occurs following cell lysis in the T3SS mutant (**Figure 1B**). *β*-lactamase was used as the reporter because of its high sensitivity, ease of operation and clear color change readout from yellow to red; however, special attention is required when using it in T3SS inhibitor screening. *β*-lactamase is not applicable when screening dark-colored compounds, as it can disturb the color readout of the reaction mixture. In addition, the positive compounds obtained using *β*-lactamase reporter need to be re-tested to avoid false positive results caused by potential lactamase-inhibitory activity.

Structure–function analysis of the BCD derivatives revealed that the single substitution at para-position on the benzene ring had the greatest effect on T3SS inhibition. It is likely that para-position substitution resulted in a stronger binding ability with the targets, or was similar to its competitors. However, although halogen substitutions of the BCD derivatives resulted in an increased T3SS inhibitory effect, it remains unknown whether other substitutions such as hydroxy, alkyl, and sulfo, also play a role in T3SS inhibition, suggesting the possible existence of other highly-efficient BCD derivatives.

The BCD compound BCD03 inhibited T3SS of plant pathogenic bacteria in distinct Hrp groups, suggesting that it targets a common component shared by T3SSs in different bacteria. Some broad-spectrum T3SS inhibitors have already been reported, such as salicylidene, acylhydrazides, and thiazolidinones, both of which affect T3SS in multiple bacterial species (Aiello et al., 2010; Duncan et al., 2012). Salicylidene acylhydrazides were first found using *Yersinia* as the screening model, and later proved effective against *Chlamydia*, *Pseudomonas*, *Escherichia*, *Salmonella* and *Shigella* bacteria (Kauppi et al., 2003; Gauthier et al., 2005; Muschiol et al., 2006; Hudson et al., 2007; Veenendaal et al., 2009). Similarly, thiazolidinone inhibits the pathogenicity of *Yersinia* by inhibiting the formation of T3SS injectisome, preventing effector secretion of *Francisella* by targeting the type IV secretion system (Felise et al., 2008). Moreover, thiazolidinone was the first T3SS inhibitor reported to inhibit the plant pathogen *P. syringae* (Felise et al., 2008). Accordingly, the inhibitory effect of BCD derivatives on animal pathogenic bacteria is an attractive subject of future study.

The discovery of T3SS inhibitors has become a significant topic in recent years, and a number of inhibitors with different action models have subsequently been examined. Salicylidene acylhydrazides derivatives have diverse inhibitory mechanisms. For example, INP0007 blocks the translocation of Yop T3Es and protects HeLa cells from *Yersinia* infection (Nordfelth et al., 2005), while INP1750 binds to YscN, the ATPase of *Yersinia*, resulting in the suppression of T3Es secretion (Swietnicki et al., 2011). Meanwhile, other inhibitors specifically target T3Es. For example, exosin, which was identified in *P. aeruginosa*, directly blocks the ADP-ribosyltransferase activity of T3E ExoS, while pseudolipasin A suppresses the phospholipase A_2_ activity of T3E ExoU (Lee et al., 2007; Arnoldo et al., 2008). Other compounds have also been screened as T3SS inhibitors, such as caminoside A, aurodox, and o-coumaric acid; however, their molecular targets remain unknown (Linington et al., 2002; Kimura et al., 2011; Fan et al., 2017). The BCD derivatives identified in this study had a significant inhibitory effect on T3Es secretion in several plant pathogenic bacteria and showed the ability to suppress the pathogenicity of *A. citrulli* on its natural host. To express sufficient T3Es for Western blotting analysis, the T3E gene *hopAO1* in *A. citrulli* and *avrXa27* gene in *X. oryzae* were placed under control of constitutive expression promoters, making it hard to distinguish between transcriptional differences by comparing Western Blotting bands. Meanwhile, expression of the T3Es gene *avrPto* under its own T3SS-inducing promoter had no obvious influence by BCD03 in terms of transcription of the reporter gene. It enhances our speculation that the BCD derivatives directly blocks T3E secretion through the T3SS complex. Our future study is to identify the potential targets of the BCD compounds in bacteria, which may help us explain their T3SS inhibitory mechanism and determine their applicability.

## Data Availability

All datasets for this study are included in the manuscript and the **Supplementary Files**.

## Author Contributions

LC, Y-NM, J-LL and L-QZ designed the research; J-LL and L-QZ supervised the study; LC and Y-NM performed all the experiments and analyzed data. Y-NM and L-QZ wrote the paper. All authors revised the manuscript and approved the final version for submission.

## Funding

This work was funded by the Key Project of Chinese National Programs for Fundamental Research and Development (973 Program) (2015CB150605), National Key Research and Development Program (2017YFD0201106), Special Fund for Agro-scientific Research in the Public Interest (201503109), and the National Natural Science Foundation of China (31572045, 31872020).

## Conflict of Interest Statement

Authors Liang Chen, Nai-Guo Si, Jun-Li Liu were employed by company Shenyang Sinochem Agrochemicals R&D Co., Ltd, Shenyang, China. The remaining authors declare that the research was conducted in the absence of any commercial or financial relationships that could be construed as a potential conflict of interest.

## References

[B1] Aiello D. Williams J. D. Majgier-Baranowska H. Patel I. Peet N. P. Huang J. (2010). Discovery and characterization of inhibitors of *Pseudomonas aeruginosa* type III secretion. Antimicrob. Agents Chemother. 54, 1988–1999. 10.1128/AAC.01598-09 20176902 PMC2863679

[B2] Alfano J. R. Bauer D. W. Milos T. M. Collmer A. (1996). Analysis of the role of the *Pseudomonas syringae* pv. *syringae* HrpZ harpin in elicitation of the hypersensitive response in tobacco using functionally non-polar *hrpZ* deletion mutations, truncated HrpZ fragments, and *hrmA* mutations. Mol. Microbiol. 19, 715–728. 10.1046/j.1365-2958.1996.415946.x 8820642

[B3] Alfano J. R. Collmer A. (1996). Bacterial pathogens in plants: life up against the wall. Plant Cell 8, 1683. 10.2307/3870222 12239358 PMC161307

[B4] Ambler R. P. (1980). The structure of β-lactamases. Philos. Trans. R. Soc. B-Biol. Sci. 289, 321–331. 10.1098/rstb.1980.0049 6109327

[B5] Anantharajah A. Mingeot-Leclercq M.-P. Van Bambeke F. (2016). Targeting the type three secretion system in *Pseudomonas aeruginosa*. Trends Pharmacol. Sci. 37, 734–749. 10.1016/j.tips.2016.05.011 27344210

[B6] Arnoldo A. Curak J. Kittanakom S. Chevelev I. Lee V. T. Sahebol-Amri M. (2008). Identification of small molecule inhibitors of *Pseudomonas aeruginosa* exoenzyme S using a yeast phenotypic screen. PLoS. Genet. 4, e1000005. 10.1371/journal.pgen.1000005 18454192 PMC2265467

[B7] Büttner D. (2012). Protein export according to schedule: architecture, assembly, and regulation of type III secretion systems from plant-and animal-pathogenic bacteria. Microbiol. Mol. Biol. Rev. 76, 262–310. 10.1128/MMBR.05017-11 22688814 PMC3372255

[B8] Badel J. L. Shimizu R. Oh H.-S. Collmer A. (2006). A *Pseudomonas syringae* pv. *tomato avrE1/hopM1* mutant is severely reduced in growth and lesion formation in tomato. Mol. Plant-Microbe Interact. 19, 99–111. 10.1094/MPMI-19-0099 16529372

[B9] Bahar O. Burdman S. (2010). Bacterial fruit blotch: a threat to the cucurbit industry. Isr. J. Plant Sci. 58, 19–31. 10.1560/IJPS.58.1.19

[B10] Bonas U. (1994). “Hrp genes of phytopathogenic bacteria,” in Bacterial pathogenesis of plants and animals. (Berlin, Heidelberg: Springer), 79–96. 10.1007/978-3-642-78624-2_4

[B11] Boucher C. A. Barberis P. A. Demery D. A. (1985). Transposon mutagenesis of *Pseudomonas solanacearum*: isolation of Tn5-induced avirulent mutants. Microbiology 131, 2449–2457. 10.1099/00221287-131-9-2449

[B12] Bowlin N. O. Williams J. D. Knoten C. A. Torhan M. C. Tashjian T. F. Li B. (2014). Mutations in the *P. aeruginosa* needle protein gene *pscF* confer resistance to phenoxyacetamide inhibitors of the type III secretion system. Antimicrob. Agents Chemother. 58, 02795–02713. 10.1128/AAC.02795-13 PMC4023729 24468789

[B13] Bretz J. R. Mock N. M. Charity J. C. Zeyad S. Baker C. J. Hutcheson S. W. (2003). A translocated protein tyrosine phosphatase of *Pseudomonas syringae* pv. *tomato* DC3000 modulates plant defence response to infection. Mol. Microbiol. 49, 389–400. 10.1046/j.1365-2958.2003.03616.x 12828637

[B14] Buell C. R. Joardar V. Lindeberg M. Selengut J. Paulsen I. T. Gwinn M. L. (2003). The complete genome sequence of the Arabidopsis and tomato pathogen *Pseudomonas syringae* pv. *tomato* DC3000 . Proc. Natl. Acad. Sci. 100, 10181–10186. 10.1073/pnas.1731982100 12928499 PMC193536

[B15] Cornelis G. R. (2006). The type III secretion injectisome. Nat. Rev. Microbiol. 4, 811. 10.1038/nrmicro1526 17041629

[B16] Diepold A. Armitage J. P. (2015). Type III secretion systems: the bacterial flagellum and the injectisome. Philos. Trans. R. Soc. B-Biol. Sci. 370, 20150020. 10.1098/rstb.2015.0020 PMC4632597 26370933

[B17] Duncan M. C. Linington R. G. Auerbuch V. (2012). Chemical inhibitors of the type three secretion system: disarming bacterial pathogens. Antimicrob. Agents Chemother. 56, 5433–5441. 10.1128/AAC.00975-12 22850518 PMC3486574

[B18] Duncan M. C. Wong W. R. Dupzyk A. J. Bray W. M. Linington R. G. Auerbuch V. (2014). An NF-κB-based high-throughput screen identifies piericidins as inhibitors of the *Yersinia pseudotuberculosis* type III secretion system. Antimicrob. Agents Chemother. 58, 1118–1126. 10.1128/AAC.02025-13 24295981 PMC3910828

[B19] Eiamphungporn W. Schaduangrat N. Malik A. Nantasenamat C. (2018). Tackling the antibiotic resistance caused by class A β-Lactamases through the use of β-Lactamase inhibitory protein. Int. J. Mol. Sci. 19, 2222. 10.3390/ijms19082222 PMC6121496 30061509

[B20] Enquist P.-A. Gylfe Hägglund U. Lindström P. Norberg-Scherman H. Sundin C. (2012). Derivatives of 8-hydroxyquinoline—antibacterial agents that target intra-and extracellular Gram-negative pathogens. Bioorg. Med. Chem. Lett. 22, 3550–3553. 10.1016/j.bmcl.2012.03.096 22525317

[B21] Fan S. Tian F. Li J. Hutchins W. Chen H. Yang F. (2017). Identification of phenolic compounds that suppress the virulence of *Xanthomonas oryzae* on rice *via the* type III secretion system. Mol. Plant Pathol. 18, 555–568. 10.1111/mpp.12415 27084974 PMC6638228

[B22] Faure E. Mear J.-B. Faure K. Normand S. Couturier-Maillard A. Grandjean T. (2014). *Pseudomonas aeruginosa* type-3 secretion system dampens host defense by exploiting the NLRC4-coupled inflammasome. Am. J. Respir. Crit. Care Med. 189, 799–811. 10.1164/rccm.201307-1358OC 24555512

[B23] Felise H. B. Nguyen H. V. Pfuetzner R. A. Barry K. C. Jackson S. R. Blanc M.-P. (2008). An inhibitor of gram-negative bacterial virulence protein secretion. Cell host Microbe. 4, 325–336. 10.1016/j.chom.2008.08.001 18854237 PMC2646588

[B24] Finan T. M. Kunkel B. De Vos G. F. Signer E. R. (1986). Second symbiotic megaplasmid in Rhizobium meliloti carrying exopolysaccharide and thiamine synthesis genes. J. Bacteriol. 167, 66–72. 10.1128/jb.167.1.66-72.1986 3013840 PMC212841

[B25] Garrity-Ryan L. K. Kim O. K. Balada-Llasat J.-M. Bartlett V. J. Verma A. K. Fisher M. L. (2010). Small molecule inhibitors of LcrF, a *Yersinia pseudotuberculosis* transcription factor, attenuate virulence and limit infection in a murine pneumonia model. Infect. Immun. 78, 4683–4690. 10.1128/IAI.01305-09 20823209 PMC2976336

[B26] Gauthier A. Robertson M. L. Lowden M. Ibarra J. A. Puente J. L. Finlay B. B. (2005). Transcriptional inhibitor of virulence factors in enteropathogenic *Escherichia coli*. Antimicrob. Agents Chemother. 49, 4101–4109. 10.1128/AAC.49.10.4101-4109.2005 16189086 PMC1251496

[B27] Grier M. C. Garrity-Ryan L. K. Bartlett V. J. Klausner K. A. Donovan P. J. Dudley C. (2010). *N*-Hydroxybenzimidazole inhibitors of ExsA MAR transcription factor in *Pseudomonas aeruginosa*: In vitro anti-virulence activity and metabolic stability. Bioorg. Med. Chem. Lett. 20, 3380–3383. 10.1016/j.bmcl.2010.04.014 20434913

[B28] Harmon D. E. Davis A. J. Castillo C. Mecsas J. (2010). Identification and characterization of small-molecule inhibitors of Yop translocation in *Yersinia pseudotuberculosis*. Antimicrob. Agents Chemother. 54, 3241–3254. 10.1128/AAC.00364-10 20498321 PMC2916352

[B29] Hopkins C. M. White F. Choi S. Guo A. Leach J. (1992). Identification of a family of avirulence genes from *Xanthomonas oryzae* pv. *oryzae*. Mol. Plant-Microbe Interact. 5, 451–459. 10.1094/MPMI-5-451 1335800

[B30] Hopkins D. Thompson C. (2002). Evaluation of Citrullus sp. germ plasm for resistance to *Acidovorax avenae* subsp. *citrulli*. Plant Dis. 86, 61–64. 10.1094/PDIS.2002.86.1.61 30823000

[B31] Hudson D. L. Layton A. N. Field T. R. Bowen A. J. Wolf-Watz H. Elofsson M. (2007). Inhibition of type III secretion in *Salmonella enterica* serovar Typhimurium by small-molecule inhibitors. Antimicrob. Agents Chemother. 51, 2631–2635. 10.1128/AAC.01492-06 17502403 PMC1913257

[B32] Jessen D. L. Bradley D. S. Nilles M. L. (2013). A type III secretion system inhibitor targets YopD while revealing differential regulation of secretion in calcium blind mutants of *Yersinia pestis*. Antimicrob. Agents Chemother. 58, 01170–01113. 10.1128/AAC.01170-13 PMC3910845 24247143

[B33] Ji Z.-Y. Xiong L. Zou L.-F. Li Y.-R. Ma W.-X. Liu L. (2014). AvrXa7-Xa7 mediated defense in rice can be suppressed by transcriptional activator-like effectors TAL6 and TAL11a from *Xanthomonas oryzae* pv. *oryzicola*. Mol. Plant-Microbe Interact. 27, 983–995. 10.1094/MPMI-09-13-0279-R 25105804

[B34] Johnson K. Minsavage G. Le T. Jones J. Walcott R. (2011). Efficacy of a nonpathogenic *Acidovorax citrulli* strain as a biocontrol seed treatment for bacterial fruit blotch of cucurbits. Plant Dis. 95, 697–704. 10.1094/PDIS-09-10-0660 30731899

[B35] Kauppi A. M. Nordfelth R. Uvell H. Wolf-Watz H. Elofsson M. (2003). Targeting bacterial virulence: inhibitors of type III secretion in *Yersinia*. Chem. Biol. 10, 241–249. 10.1016/S1074-5521(03)00046-2 12670538

[B36] Keane P. Kerr A. New P. (1970). Crown gall of stone fruit II. Identification and nomenclature of *Agrobacterium* isolates. Aust. J. Biol. Sci. 23, 585–596. 10.1071/BI9700585

[B37] Keyser P. Elofsson M. Rosell S. Wolf-Watz H. (2008). Virulence blockers as alternatives to antibiotics: type III secretion inhibitors against Gram-negative bacteria. J. Intern. Med. 264, 17–29. 10.1111/j.1365-2796.2008.01941.x 18393958

[B38] Kim D. Baek J. Song J. Byeon H. Min H. Min K. H. (2014). Identification of arylsulfonamides as ExoU inhibitors. Bioorg. Med. Chem. Lett. 24, 3823–3825. 10.1016/j.bmcl.2014.06.064 25027940

[B39] Kim O. K. Garrity-Ryan L. K. Bartlett V. J. Grier M. C. Verma A. K. Medjanis G. (2009). *N*-hydroxybenzimidazole inhibitors of the transcription factor LcrF in *Yersinia*: novel antivirulence agents. J. Med. Chem. 52, 5626–5634. 10.1021/jm9006577 19708663 PMC2778250

[B40] Kimura K. Iwatsuki M. Nagai T. Matsumoto A. Takahashi Y. Shiomi K. (2011). A small-molecule inhibitor of the bacterial type III secretion system protects against *in vivo* infection with *Citrobacter rodentium*. J. Antibiot. 64, 197. 10.1038/ja.2010.155 21139624

[B41] Kovach M. Phillips R. Elzer P. Peterson K. (1994). pBBR1MCS: a broad-host-range cloning vector. Biotechniques 16, 800–802. 10.1002/bip.360340510 8068328

[B42] Lee V. T. Pukatzki S. Sato H. Kikawada E. Kazimirova A. A. Huang J. (2007). Pseudolipasin A is a specific inhibitor for phospholipase A2 activity of *Pseudomonas aeruginosa* cytotoxin ExoU. Infect. Immun. 75, 1089–1098. 10.1128/IAI.01184-06 17178785 PMC1828555

[B43] Li X. Kang F.-A. Macielag M. (2005). “Triazine compounds as inhibitors of bacterial type III protein secretion systems”. Google Patents.

[B44] Lidell M. Hutcheson S. (1994). Characterization of the *hrpJ* and *hrpU* operons of *Pseudomonas syringae* pv. *syringae* Pss61: similarity with components of enteric bacteria involved in flagellar biogenesis and demonstration of their role in HarpinPss secretion. Mol. Plant-Microbe Interact. 7, 488–497. 10.1094/MPMI-7-0488 8075421

[B45] Linington R. G. Robertson M. Gauthier A. Finlay B. B. Van Soest R. Andersen R. J. (2002). Caminoside A, an antimicrobial glycolipid isolated from the marine sponge *Caminus sphaeroconia*. Org. Lett. 4, 4089–4092. 10.1021/ol0268337 12423093

[B46] Liu X. Zou H. Zou L. Chen G. (2010). Site-directed mutagenesis and functional analysis of *hrcU* gene from rice pathogen *Xanthomonas oryzae* pv. *oryzae*. Chin. J. Rice Sci. 24, 348–352. 10.3724/SP.J.1011.2010.01385

[B47] Luo Z.-Q. Farrand S. K. (1999). Signal-dependent DNA binding and functional domains of the quorum-sensing activator TraR as identified by repressor activity. Proc. Natl. Acad. Sci. 96, 9009–9014. 10.1073/pnas.96.16.9009 10430886 PMC17723

[B48] Lynch S. V. Flanagan J. L. Sawa T. Fang A. Baek M. S. Rubio-Mills A. (2010). Polymorphisms in the *Pseudomonas aeruginosa* type III secretion protein, PcrV — implications for anti-PcrV immunotherapy. Microb. Pathog. 48, 197–204. 10.1016/j.micpath.2010.02.008 20211240 PMC2860055

[B49] Marsden A. E. King J. M. Spies M. A. Kim O. K. Yahr T. L. (2015). Inhibition of *Pseudomonas aeruginosa* ExsA DNA-binding activity by *N*-hydroxybenzimidazoles. Antimicrob. Agents Chemother. 60, 02242–02215. 10.1128/AAC.02242-15 PMC4750669 26574012

[B50] Marsden A. E. King J. M. Spies M. A. Kim O. K. Yahr T. L. (2016). Inhibition of *Pseudomonas aeruginosa* ExsA DNA-binding activity by *N*-hydroxybenzimidazoles. Antimicrob. Agents Chemother. 60, 766–776. 10.1128/AAC.02242-15 26574012 PMC4750669

[B51] Matagne A. Dubus A. Galleni M. Frère J.-M. (1999). The β-lactamase cycle: a tale of selective pressure and bacterial ingenuity. Nat. Prod. Rep. 16, 1–19. 10.1039/a705983c 10101880

[B52] Muschiol S. Bailey L. Gylfe Å. Sundin C. Hultenby K. Bergström S. (2006). A small-molecule inhibitor of type III secretion inhibits different stages of the infectious cycle of *Chlamydia trachomatis*. Proc. Natl. Acad. Sci. 103, 14566–14571. 10.1073/pnas.0606412103 16973741 PMC1566191

[B53] Nilles M. L. Condry D. L. J. (2017). Type 3 Secretion Systems. (New York, NY: Springer). 10.1007/978-1-4939-6649-3

[B54] Nordfelth R. Kauppi A. M. Norberg H. Wolf-Watz H. Elofsson M. (2005). Small-molecule inhibitors specifically targeting type III secretion. Infect. Immun. 73, 3104–3114. 10.1128/IAI.73.5.3104-3114.2005 15845518 PMC1087345

[B55] Pan N. Goguen J. Lee C. (2007). “High throughput screening for small-molecule inhibitors of type III secretion in *Yersinia pestis*,” in The Genus Yersinia. (New York, NY: Springer) 367–375. 10.1007/978-0-387-72124-8_34 17966433

[B56] Pan N. J. Brady M. J. Leong J. M. Goguen J. D. (2009). Targeting type III secretion in *Yersinia pestis*. Antimicrob. Agents Chemother. 53, 385–392. 10.1128/AAC.00670-08 19015348 PMC2630656

[B57] Rasko D. A. Sperandio V. (2010). Anti-virulence strategies to combat bacteria-mediated disease. Nat. Rev. Drug Discov. 9, 117. 10.1038/nrd3013 20081869

[B58] Ren Z. Jiang W. Ni X. Lin M. Zhang W. Tian G. (2014). Multiplication of *Acidovorax citrulli* in planta during infection of melon seedlings requires the ability to synthesize leucine. Plant Pathol. 63, 784–791. 10.1111/ppa.12156

[B59] Schaad N. W. Postnikova E. Sechler A. Claflin L. E. Vidaver A. K. Jones J. B. (2008). Reclassification of subspecies of *Acidovorax avenae* as *A. Avenae* (Manns 1905) emend., *A. cattleyae* (Pavarino, 1911) comb. nov., *A. citrulli* (Schaad et al., 1978) comb. nov., and proposal of *A. oryzae* sp. nov. Syst. Appl. Microbiol. 31, 434–446. 10.1016/j.syapm.2008.09.003 18993005

[B60] Sebaugh J. (2011). Guidelines for accurate EC50/IC50 estimation. Pharm. Stat. 10, 128–134. 10.1002/pst.426 22328315

[B61] Swietnicki W. Carmany D. Retford M. Guelta M. Dorsey R. Bozue J. (2011). Identification of small-molecule inhibitors of *Yersinia pestis* type III secretion system YscN ATPase. PLoS One 6, e19716. 10.1371/journal.pone.0019716 21611119 PMC3097197

[B62] Tampakaki A. P. Skandalis N. Gazi A. D. Bastaki M. N. Panagiotis F, S. Charova S. N. (2010). Playing the “Harp”: evolution of our understanding of *hrp*/*hrc* genes. Annu. Rev. Phytopathol. 48, 347–370. 10.1146/annurev-phyto-073009-114407 20455697

[B63] Tsuge S. Furutani A. Fukunaka R. Takashi O. Tsuno K. Ochiai H. (2002). Expression of *Xanthomonas oryzae* pv. *oryzae hrp* genes in XOM2, a novel synthetic medium. J. Gen. Plant Pathol. 68, 363–371. 10.1007/PL00013104

[B64] Underwood W. Zhang S. He S. Y. (2007). The *Pseudomonas syringae* type III effector tyrosine phosphatase HopAO1 suppresses innate immunity in *Arabidopsis thaliana*. Plant J. 52, 658–672. 10.1111/j.1365-313X.2007.03262.x 17877704

[B65] Veenendaal A. K. Sundin C. Blocker A. J. (2009). Small-molecule type III secretion system inhibitors block assembly of the *Shigella* type III secreton. Bacteriol. 191, 563–570. 10.1128/JB.01004-08 PMC2620818 18996990

[B66] Wang D. Zetterstrom C. E. Gabrielsen M. Beckham K. S. Tree J. J. Macdonald S. E. (2011). Identification of bacterial target proteins for the salicylidene acylhydrazide class of virulence blocking compounds. J. Biol. Chem. M111, 233858. 10.1074/jbc.M111.233858 PMC3191033 21724850

[B67] Warrener P. Varkey R. Bonnell J. C. Digiandomenico A. Camara M. Cook K. (2014). A novel anti-PcrV antibody providing enhanced protection against *Pseudomonas aeruginosa* in multiple animal infection models. Antimicrob. Agents Chemother. 58, 4384–4391. 10.1128/AAC.02643-14 24841258 PMC4136058

[B68] Wei H.-L. Chakravarthy S. Mathieu J. Helmann T. C. Stodghill P. Swingle B. (2015). *Pseudomonas syringae* pv. *tomato* DC3000 type III secretion effector polymutants reveal an interplay between HopAD1 and AvrPtoB. Cell host Microbe 17, 752–762. 10.1016/j.chom.2015.05.007 26067603 PMC4471848

[B69] Wengelnik K. Bonas U. (1996). HrpXv, an AraC-type regulator, activates expression of five of the six loci in the hrp cluster of *Xanthomonas campestris* pv. *vesicatoria*. J. Bacteriol. 178, 3462–3469. 10.1111/j.1365-2672.1996.tb03274.x 8655542 PMC178114

[B70] Wengelnik K. Van Den Ackerveken G. Bonas U. (1996). HrpG, a key hrp regulatory protein of *Xanthomonas campestris* pv. *vesicatoria* ls homologous to two-component response regulators. Mol. Plant-Microbe Interact. 9, 704–712. 10.1094/MPMI-9-0704 8870269

[B71] Williams J. D. Torhan M. C. Neelagiri V. R. Brown C. Bowlin N. O. Di M. (2015). Synthesis and structure–activity relationships of novel phenoxyacetamide inhibitors of the *Pseudomonas aeruginosa* type III secretion system (T3SS). Bioorg. Med. Chem. 23, 1027–1043. 10.1016/j.bmc.2015.01.011 25638499 PMC4339527

[B72] Yamazaki A. Li J. Zeng Q. Khokhani D. Hutchins W. C. Yost A. C. (2012). Derivatives of plant phenolic compound affect the type III secretion system of *Pseudomonas aeruginosa via a* GacS-GacA two-component signal transduction system. Antimicrob. Agents Chemother. 56, 36–43. 10.1128/AAC.00732-11 21968370 PMC3256035

[B73] Yang F. Korban S. S. Pusey P. L. Elofsson M. Sundin G. W. Zhao Y. (2014). Small-molecule inhibitors suppress the expression of both type III secretion and amylovoran biosynthesis genes in *Erwinia amylovora*. Mol. Plant Pathol. 15, 44–57. 10.1111/mpp.12064 23915008 PMC6638656

[B74] Zhang X. Zhao M. Yan J. Yang L. Yang Y. Guan W. (2018). Involvement of *hrpX* and *hrpG* in the virulence of *Acidovorax citrulli* strain Aac5, causal agent of bacterial fruit blotch in cucurbits. Front. Microb. 9, 507. 10.3389/fmicb.2018.00507 PMC5880930 29636729

