# Supplementary material for: Identification of Benzyloxy Carbonimidoyl Dicyanide Derivatives as Novel Type III Secretion System Inhibitors via High-Throughput Screening
Source: Front Plant Sci. 2019 Sep 5;10:1059. doi: 10.3389/fpls.2019.01059 (PMC6739442; doi:10.3389/fpls.2019.01059)
Supplement: Supplementary file 1 [file Image_1.pdf]

Identification of Benzyloxy Carbonimidoyl Dicyanide Derivatives as Novel Type III Secretion System Inhibitors via High-Throughput Screening

Yi-Nan Ma[1†], Liang Chen[1, 2†], Nai-Guo Si[2], Wen-Jun Jiang[1], Zhi-Gang Zhou[3], Jun-Li Liu[2], Li-Qun Zhang[1*]

[1]Department of Plant Pathology and MOA Key Laboratory of Pest Monitoring and Green Management, China Agricultural University, Beijing, China;

[2]State Key Laboratory of the Discovery and Development of Novel Pesticide, Shenyang Sinochem Agrochemicals R&D Co., Ltd, Shenyang, China.

[3]China-Norway Joint Lab on Fish Gut Microbiota, Feed Research Institute, Chinese Academy of Agricultural Sciences, Beijing 100081, PR China.

***Corresponding author:**
Li-Qun Zhang,
zhanglq@cau.edu.cn

[†]These authors have contributed equally to this work

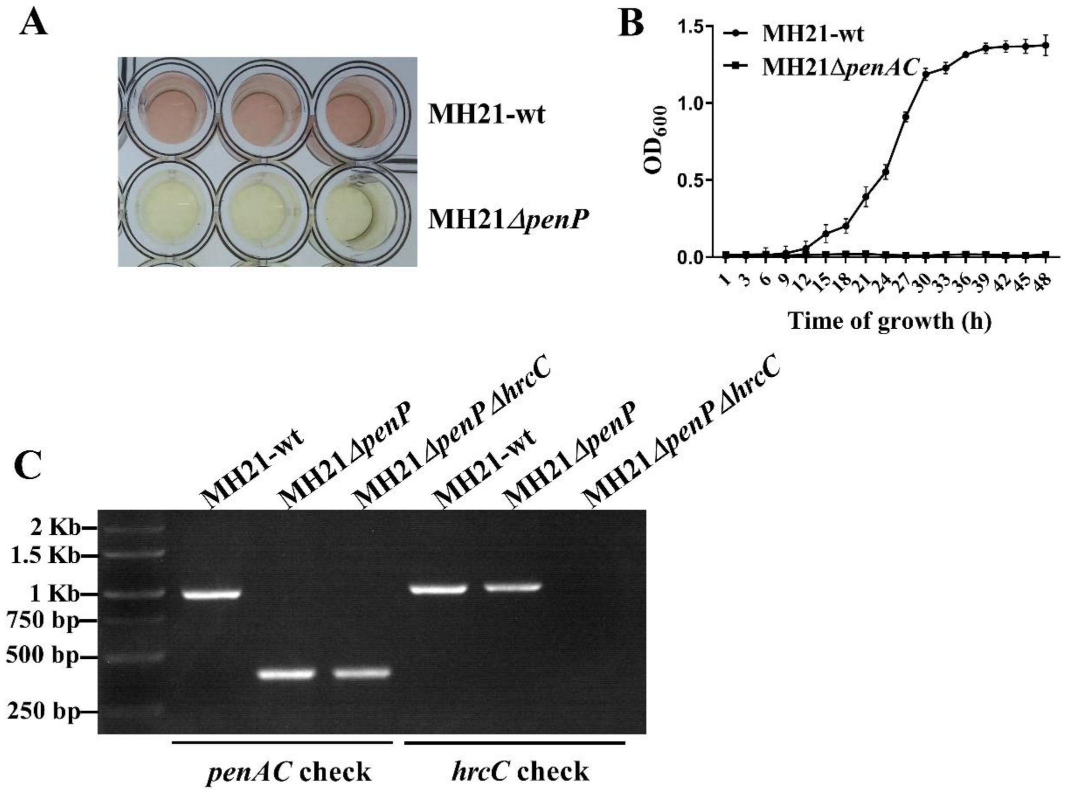

Figure S1. Mutant construction and functional analysis of the *penAC* gene in *Acidovorax citrulli* MH21. (A) Bacterial suspension of MH21 wild-type and mutant Δ*penAC*. Photographs were taken 15 min after supplementation of nitrocefin (20 μg/mL). (B) Growth curves of MH21 wild-type and mutant Δ*penAC* in LB medium supplemented with 50 μg/mL ampicillin (Ap). (C) MH21 wild-type, mutant Δ*penAC* and double mutantΔ*penAC*Δ*hrcC* were confirmed by PCR amplification using *penAC*-dF/-dR or *hrcC*-dF/-dR primers, respectively.
