# Supplementary material for: Identification of Benzyloxy Carbonimidoyl Dicyanide Derivatives as Novel Type III Secretion System Inhibitors via High-Throughput Screening
Source: Front Plant Sci. 2019 Sep 5;10:1059. doi: 10.3389/fpls.2019.01059 (PMC6739442; doi:10.3389/fpls.2019.01059)
Supplement: Supplementary file 3 [file Image_3.pdf]

Identification of Benzyloxy Carbonimidoyl Dicyanide Derivatives as Novel Type III Secretion System Inhibitors via High-Throughput Screening

Yi-Nan Ma[1†], Liang Chen[1, 2†], Nai-Guo Si[2], Wen-Jun Jiang[1], Zhi-Gang Zhou[3], Jun-Li Liu[2], Li-Qun Zhang[1*]

[1]Department of Plant Pathology and MOA Key Laboratory of Pest Monitoring and Green Management, China Agricultural University, Beijing, China;

[2]State Key Laboratory of the Discovery and Development of Novel Pesticide, Shenyang Sinochem Agrochemicals R&D Co., Ltd, Shenyang, China.

[3]China-Norway Joint Lab on Fish Gut Microbiota, Feed Research Institute, Chinese Academy of Agricultural Sciences, Beijing 100081, PR China.

***Corresponding author:**
Li-Qun Zhang,
zhanglq@cau.edu.cn

[†]These authors have contributed equally to this work

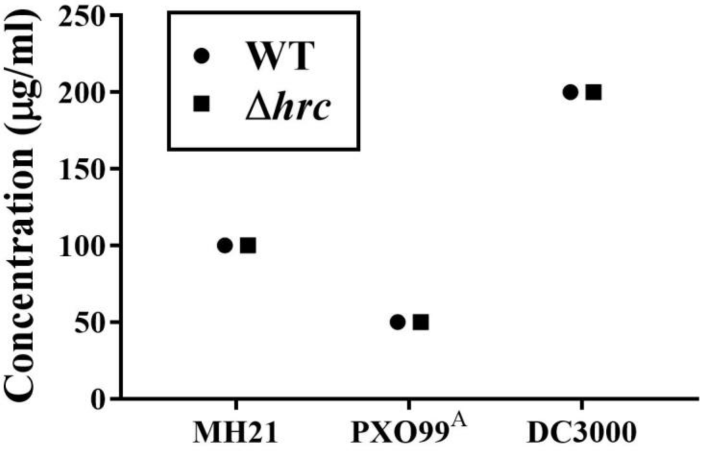

Figure S3. Minimum inhibitory concentrations (MICs) of MH21, PXO99^{A}, DC3000 and their T3SS mutants (MH21Δ*hrcC*, PXO99^{A}Δ*hrcU* and DC3000Δ*hrcQ-U*).
