# Supplementary material for: Identification of Benzyloxy Carbonimidoyl Dicyanide Derivatives as Novel Type III Secretion System Inhibitors via High-Throughput Screening
Source: Front Plant Sci. 2019 Sep 5;10:1059. doi: 10.3389/fpls.2019.01059 (PMC6739442; doi:10.3389/fpls.2019.01059)
Supplement: Supplementary file 4 [file Table_1.pdf]

# Identification of Benzyloxy Carbonimidoyl Dicyanide Derivatives as Novel Type III Secretion System Inhibitors via High-Throughput Screening

**Yi-Nan Ma[1†], Liang Chen[1, 2†], Nai-Guo Si[2], Wen-Jun Jiang[1], Zhi-Gang Zhou[3], Jun-Li Liu[2], Li-Qun Zhang[1]***

[1]Department of Plant Pathology and MOA Key Laboratory of Pest Monitoring and Green Management, China Agricultural University, Beijing, China;

[2]State Key Laboratory of the Discovery and Development of Novel Pesticide, Shenyang Sinochem Agrochemicals R&D Co., Ltd, Shenyang, China.

[3]China-Norway Joint Lab on Fish Gut Microbiota, Feed Research Institute, Chinese Academy of Agricultural Sciences, Beijing 100081, PR China.

***Corresponding author:**
Li-Qun Zhang,
zhanglq@cau.edu.cn

**[†]These authors have contributed equally to this work**

Table S1. Primers used in this study.

| Primer | Sequence (5'- 3') | Description |
|---|---|---|
| *penAC*-F | ATGAATTCGGCAAGAAATCCATGGGCTCC | Amplifies *penAC* without the 5' signal peptide sequence. |
| *penAC*-R | ATGTCGACGCCCCTGACAGCGGGGGCTG | |
| Aave-3502sig-F | ATCATATGCCGCACGTTTCATTGGCATG | Amplifies the 5' signal peptide sequence of *Aave_3502*. |
| Aave-3502sig-R | ATGAATTCCCTGCCTGGGTCGAATGCCGGA | |
| *penAC*-dF | ATGGCAAGAAATCCATGGGCTCC | *penAC* mutant detection. |
| *penAC*-dR | ATGCCCCTGACAGCGGGGGCTG | |
| *hrcC*-dF | GGATCTCGGTCTTCTTGACGAGG | *hrcC* mutant detection. |
| *hrcC*-dR | ATCAAGGGATTCAGCCGCGAC | |

Table S2. The three equivalent structural and functional elements of class A β-lactamase.

| Strain | Ambler class | $Ser^{70}$-X-X-$Lys^{73}$ | $Ser^{130}$-X-$Asn^{132}$ | $Lys^{234}$-Thr/Ser-Gly |
|---|---|---|---|---|
| *Bacillus licheniformis* BlaP | A | ---S T F K--- | ---S D N--- | ---K T G--- |
| *Escherichia coli* pC15-1a TEM-1 | A | ---S T F K--- | ---S D N--- | ---K S G--- |
| *Escherichia coli* HB101 SHV-1 | A | ---S T F K--- | ---S D N--- | ---K T G--- |
| *Serratia marcescens* S6 SME-1 | A | ---S S F K--- | ---S D N--- | ---K T G--- |
| *Acidovorax citrulli* PenAC | A | ---S T I K--- | ---S D N--- | ---K T G--- |
| *Bacillus subtilis* PenP | A | ---S T Y K--- | ---S D N--- | ---K S G--- |
| *Staphylococcus* aureus PC1 | A | ---S T S K--- | ---S D N--- | ---K S G--- |
| *Pseudomonas aeruginosa* PAO1 AmpC | C | ---S V S K--- | ---Q V F--- | ---L L N--- |
| *Bacillus cereus* BcII | B | ---Q L N K--- | ---F K K--- | ---C L V--- |
| *Acinetobacter baumannii* OXA-58 | D | ---D G Q N--- | ---G E A--- | ---S G W--- |

Table S3. $OD_{600}$ indicating the minimal inhibitory concentration (MIC) of *A. citrulli*△*penAC*(pZAC-3502sig-*penAC*) treated with BCD derivatives.

| BCD derivative | Concentration (μg/mL) | | | | | | | | | MIC (μg/mL) |
|---|---|---|---|---|---|---|---|---|---|---|
| | 250 | 200 | 150 | 100 | 75 | 50 | 25 | 12.5 | 6.25 | |
| BCD01 | 0.053 | 0.062 | 0.058 | 0.049 | 0.062 | 0.616 | 0.681 | 0.698 | 0.700 | 75 |
| BCD02 | 0.079 | 0.099 | 0.054 | 0.088 | 0.085 | 0.099 | 0.641 | 0.660 | 0.667 | 50 |
| BCD03 | 0.078 | 0.056 | 0.059 | 0.080 | 0.577 | 0.638 | 0.662 | 0.677 | 0.733 | 100 |
| BCD04 | 0.089 | 0.058 | 0.091 | 0.095 | 0.063 | 0.628 | 0.683 | 0.769 | 0.723 | 75 |
| BCD05 | 0.081 | 0.061 | 0.071 | 0.079 | 0.074 | 0.051 | 0.558 | 0.621 | 0.639 | 50 |
| BCD06 | 0.032 | 0.033 | 0.035 | 0.039 | 0.049 | 0.058 | 0.509 | 0.588 | 0.614 | 50 |
| BCD07 | 0.039 | 0.038 | 0.043 | 0.043 | 0.041 | 0.540 | 0.626 | 0.618 | 0.685 | 75 |
| BCD08 | 0.055 | 0.058 | 0.062 | 0.040 | 0.043 | 0.051 | 0.598 | 0.656 | 0.639 | 50 |
| BCD09 | 0.040 | 0.058 | 0.062 | 0.055 | 0.042 | 0.052 | 0.522 | 0.595 | 0.629 | 50 |
| BCD10 | 0.071 | 0.069 | 0.054 | 0.057 | 0.557 | 0.593 | 0.652 | 0.690 | 0.689 | 100 |
| BCD11 | 0.031 | 0.036 | 0.038 | 0.056 | 0.058 | 0.639 | 0.646 | 0.613 | 0.672 | 75 |
| BCD12 | 0.089 | 0.062 | 0.079 | 0.078 | 0.581 | 0.609 | 0.640 | 0.643 | 0.688 | 100 |
| BCD13 | 0.035 | 0.036 | 0.047 | 0.043 | 0.057 | 0.639 | 0.648 | 0.644 | 0.736 | 75 |
| BCD14 | 0.092 | 0.094 | 0.092 | 0.047 | 0.054 | 0.349 | 0.636 | 0.703 | 0.754 | 75 |

BCD01-14 are annotated in Table 2 and Figure 2B.
